# Potato growth, nitrogen balance, quality, and productivity response to water-nitrogen regulation in a cold and arid environment

**DOI:** 10.3389/fpls.2024.1451350

**Published:** 2024-10-16

**Authors:** Dandan Su, Hengjia Zhang, Anguo Teng, Changlong Zhang, Lian Lei, Yuchun Ba, Chenli Zhou, Fuqiang Li, Xietian Chen, Zeyi Wang

**Affiliations:** ^1^ College of Agriculture and Biology, Liaocheng University, Liaocheng, China; ^2^ College of Water Conservancy and Hydropower Engineering, Gansu Agricultural University, Lanzhou, China; ^3^ Yimin Irrigation Experimental Station, Minle, China

**Keywords:** potato growth, yield, quality, nitrogen balance, irrigation water use efficiency, nitrogen use efficiency, cold and arid area

## Abstract

**Background:**

The pervasively imprudent practices of irrigation and nitrogen (N) application within Oasis Cool Irrigation zones have led to significant soil nitrogen loss and a marked decrease in water and nitrogen use efficiency.

**Methods:**

To address this concern, a comprehensive field experiment was conducted from April to September in 2023 to investigate the impact of varying degrees of water and fertilization regulation strategies on pivotal parameters including potato yield, quality, nitrogen balance, and water-nitrogen use efficiency. The experimental design incorporated two water deficit degrees at potato seedling (W1, 55%-65% of Field Capacity (FC); W2, 45%-55% of FC), and four distinct nitrogen application gradients (N0, 0 kg ha-1 of N; N1, 130 kg ha-1 of N; N2, 185 kg ha-1 of N; N3, 240 kg ha-1 of N). A control was also included, comprising N0 nitrogen application and full irrigation (W0, 65%-75% of FC), totally eight treatments and one check.

**Results:**

The results indicated that the tuber yield, plant dry matter accumulation, plant height, plant stem, and leaf area index increased with higher nitrogen fertilizer application and irrigation volume. However, tuber starch content, vitamin C, and protein content initially increased and then decreased, while reducing sugar content consistently decreased. Except for W1N2 treatment, the irrigation water use efficiency increased as the N application rate rose, while the nitrogen partial factor productivity, crop nitrogen use efficiency and soil nitrogen use efficiency decreased with an increase in N fertilizer application. The W1N2 treatment resulted in a higher yield (43.16 t ha-1), highest crop nitrogen use efficiency (0.95) and systematic nitrogen use efficiency (0.72),while maintaining moderate levels of soil nitrate and ammonium nitrogen.

**Conclusion:**

Therefore, through the construction of an integrated evaluation index (IEI), the W1N2 treatment of mild water deficit (55%-65% of FC) at potato seedling combined with the medium nitrogen application (185 kg ha-1 of N) has the highest IEI (0.978), it was recommended as the optimal water-nitrogen regulation and management strategies to facilitate high-yield, high-efficiency, and environmentally sustainable potato production in the cold and arid oasis areas of northwest China.

## 1 Introduction

The potato (*Solanum tuberosum L*.), ranked as the fourth largest food crop globally after wheat, corn, and rice, plays a crucial role in ensuring global food security. Potatoes are expected to contribute significantly to China’s future food production increases (Koch et al., 2020; CIP, 2020). The northwest region of China, with its unique environmental and soil characteristics, has emerged as the primary potato producing area. However, the Oasis Irrigation Region, a key agricultural zone in northwest China, has been increasingly impacted by global warming. The accelerated melting of glaciers and retreat of snowlines have led to reduced river inflows and intensified extreme climatic events (Chen et al., 2016). These changes underscore the critical role of water resources in sustainable agriculture for the region. Moreover, the prevalent “high input, high output” approach among farmers has resulted in excessive fertilizer use, reducing fertilizer efficiency and exacerbating water resource shortages, thereby affecting the sustainable use of farmland (Cui et al., 2018; Wang et al., 2019; Pan et al., 2023).

Drought can significantly impact crop yield and quality by disrupting growth and development, which in turn affects economic returns for growers (Garg et al., 2022). Potatoes, with their shallow root systems concentrated in the top 10-30 cm of soil, are particularly sensitive to water and fertilizer management (Ferreira et al., 2017; Wu et al., 2022). Various irrigation strategies, methods, and water saving technologies can greatly influence crop yield and quality (Fandika et al., 2016; El-Abedin et al., 2017; Martinez-Romero et al., 2019). Plants respond differently to water deficits at various growth stages, with water stress before the reproductive stage potentially inhibiting aboveground growth but possibly leading to yield compensation after rehydration (Wagg et al., 2021). Conversely, moderate water deficit in later growth stages can enhance tuber quality by improving soil aeration (Fang et al., 2023). Effective irrigation practices positively affect aboveground growth parameters such as plant height, stem thickness, and leaf area index, thereby improving dry matter accumulation and transportation and enhancing yield (Xing et al., 2022). However, excessive irrigation can waste water, reduce soil oxygen levels, increase salt concentration, and cause root and tuber issues, leading to decreased yield and quality and environmental pollution (Jiang et al., 2023a; Ravensbergen et al., 2023).

Fertilizers are crucial for promoting potato growth and achieving high yields, but overuse impedes sustainable agricultural practices (Huang et al., 2021; Ren et al., 2022). Nitrogen fertilizers, in particular, pose a risk of nitrogen loss when applied beyond plant needs (Ning et al., 2017; Makani et al., 2020; Bohman et al., 2021a). Given the fragile ecological environment of the oasis irrigation area, it is essential to develop suitable water and nitrogen regulation strategies that fit local conditions. Irrigation is vital for maintaining stable and high potato yields in the Hexi oasis region. Traditional irrigation methods, such as flood and furrow irrigation, have become less favorable due to high water consumption, significant seepage, and low efficiency. Drip irrigation, adapted to the region’s flat topography and good irrigation conditions, has been introduced. During the summer growing season, film mulching has gained popularity due to high evaporation rates. The combination of drip irrigation and film mulching is being increasingly adopted to address water shortages and excessive fertilization (Gao et al., 2017; Yang et al., 2017; Yan et al., 2020).

Previous studies have primarily focused on fertilizer input, yield, and economic benefits, including factors like yield, N partial factor productivity (NPFP), water use efficiency (WUE), and economic returns (Wang et al., 2020; Wu et al., 2022; Yang et al., 2023b). However, assessing nitrogen use efficiency (NUE) through NPFP has often overlooked indigenous nitrogen supply (INS), potentially leading to overestimations of NUE in regions with soil nitrogen accumulation (Congreves et al., 2021). There is also a lack of research on water and nitrogen management in potato cultivation within cold and arid irrigation regions. We hypothesize that formulating an optimal water and nitrogen regulation strategy could effectively balance crop growth, nitrogen uptake, soil inorganic nitrogen content, and overall water and nitrogen use efficiency. The objectives of this study were to: (1) evaluate the impacts of different irrigation levels and nitrogen fertilizer applications on potato growth, yield, quality, plant N uptake, soil inorganic N content, N balance, irrigation water use efficiency (IWUE), and NUE; and (2) assess NUE from multiple perspectives and develop an integrated evaluation index (IEI) to identify an optimal water and nitrogen regulation strategy for high yield, quality, and environmental sustainability.

## 2 Materials and methods

### 2.1 Experimental site

**Figure 1:**
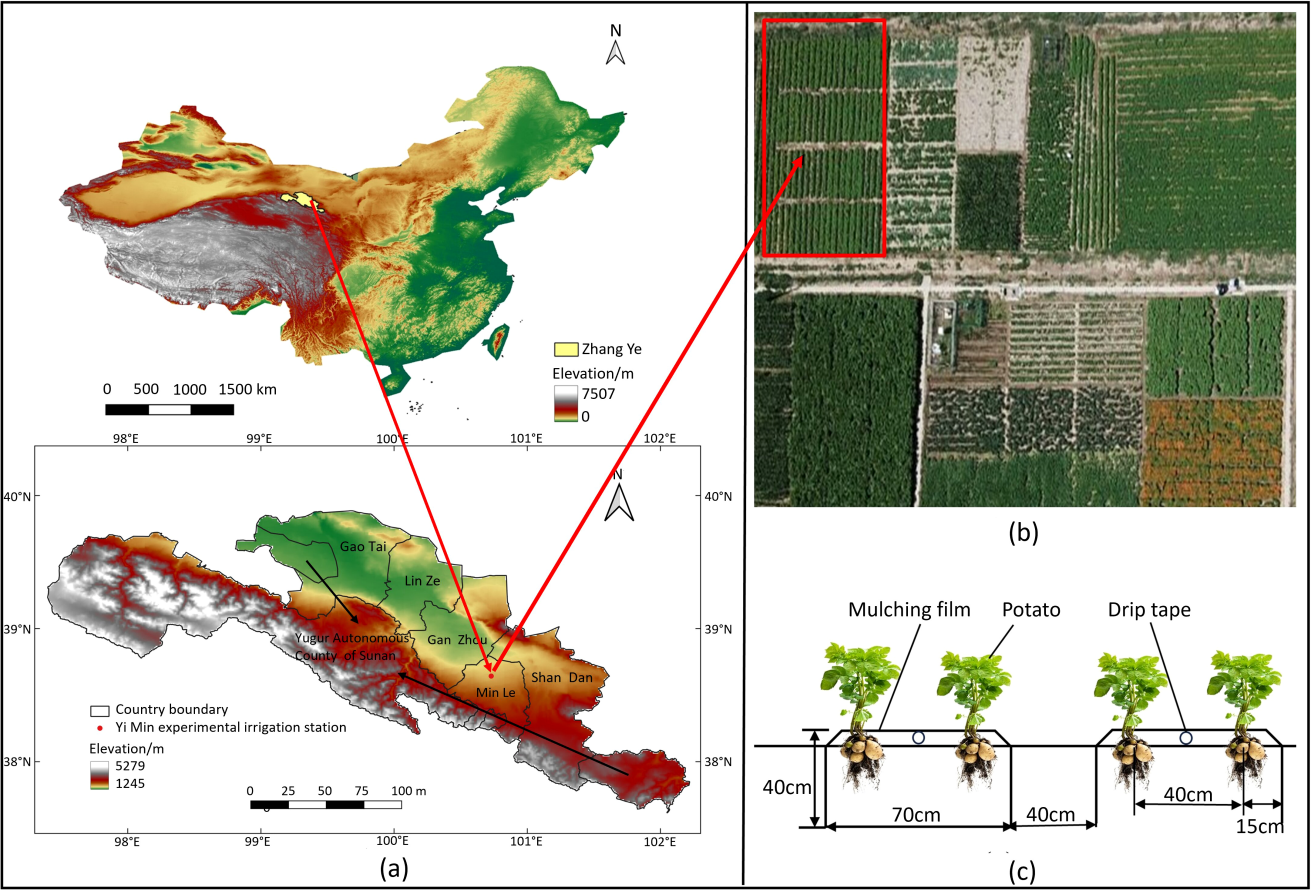
Location of the experimental site and the experimental deployment. **(A)** Geographical positioning of the experimental site; **(B)** The layout of the potato experimental field; and **(C)** the configuration of the drip irrigation belts.

**Figure 2:**
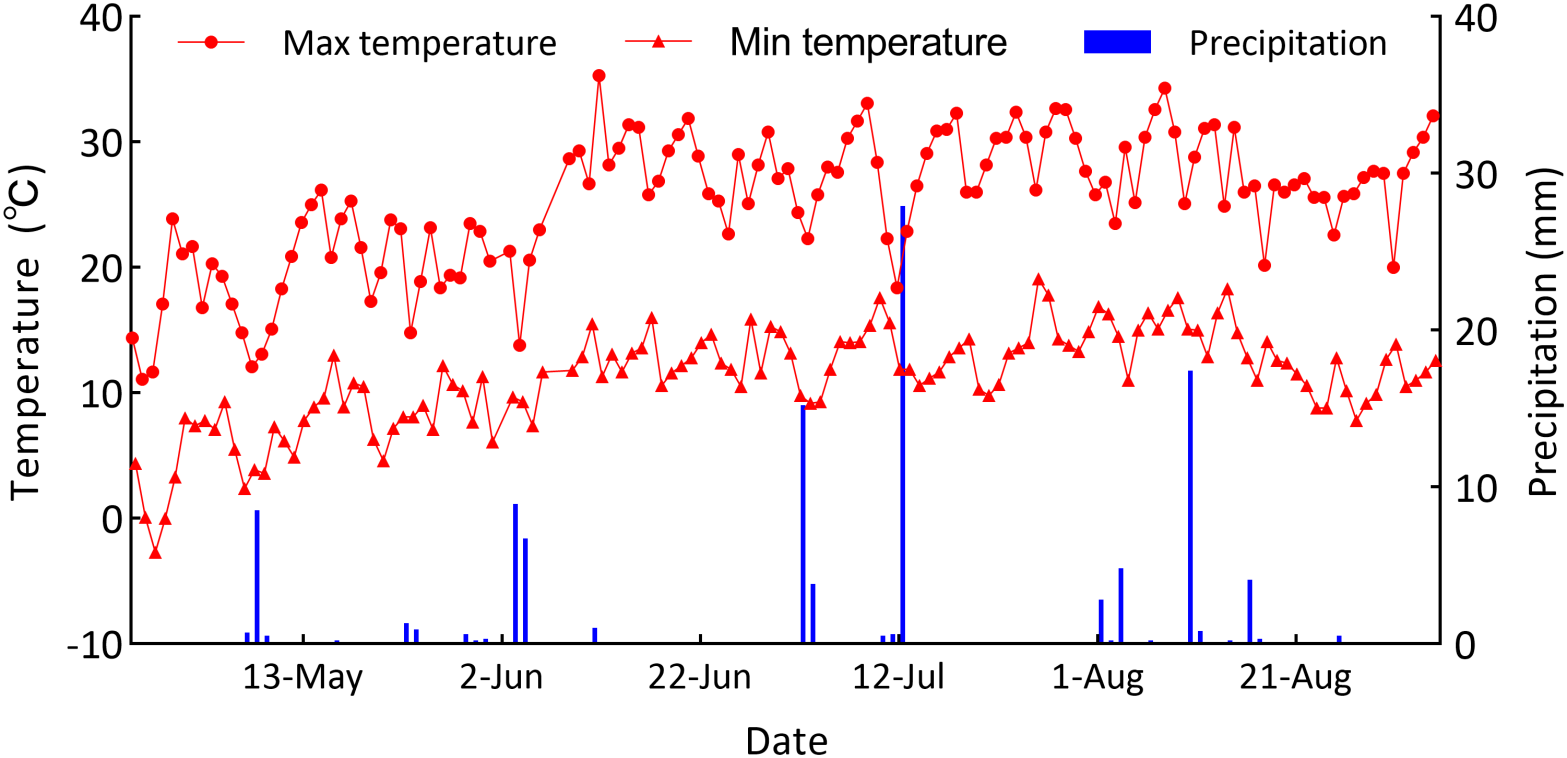
Precipitation, daily maximum and minimum temperature in the study area from April to September.

The field experiment was conducted from April to September 2023 at the Yimin Irrigation Experimental Station in Minle County, Gansu Province of China, which was located in Sanbao Town of Minle County (100°43′E, 38°39′N, 1970m a.s.l.), a cold and arid area in Hexi oasis irrigation region (**Figure 1A**). The study area was a continental desert grassland climate, with the annual precipitation mostly ranging between 183-345 mm, the average annual temperature of approximately 7.6°C, annual evaporation of about 2,000 mm, an average annual sunshine duration of about 3,000 hours, and the frost-free period of about 165 days per year. In the experimental year the region experienced a drought climate, with the total precipitation during the potato growing season of 109.6 mm and the effective precipitation (single precipitation >5 mm) of only 84.6 mm while the average temperature of 16.51°C, as was shown in **Figure 2**. The soil in the experimental site was light loam, with the basic physical and chemical properties in the 0-60 cm plow soil layer before sowing being as follows: the field capacity (FC) was 24% (gravimetrically measured), the soil bulk density was 1.46 g cm^-3^ (0-20: 1.32 g cm^-3^, 20-40: 1.44 g cm^-3^, 40-60: 1.62 g cm^-3^), the pH value was 7.84, while the organic matter content, total nitrogen content, available phosphorus content, and available potassium content were 11.8 g kg^-1^, 0.62 g kg^-1^, 15.7 mg kg^-1^, and 188.6 mg kg^-1^ respectively. The groundwater level was below 20 m, and there was no salinization phenomenon.

### 2.2 Experimental design

This study employed a two-factor experimental design, using the irrigation level serving as the primary factor and the nitrogen application rate as the secondary factor. Three water deficit and four nitrogen application levels were designed, totally eight water-nitrogen regulation treatments and a control (CK, with sufficient irrigation combined with no nitrogen fertilizer) were established. According to the growth characteristics of potatoes, the plant growth period was divided into four stages: seedling (from April 26 to June 4, totally 40 days), tuber formation (from June 5 to June 29, totally 25 days), tuber expansion (from June 30 to July 29, totally 30 days), and starch accumulation (from July 30 to September 4, totally 35 days). Considering the arid conditions of the northwestern inland river basins, where low rainfall and high evaporation are prevalent, the plants were subjected to mild water deficit (W1, 55%-65% of FC) or moderate water deficit (W2, 45%-55% of FC) at potato seedling, while sufficient irrigation (W0, 65%-75% of FC) was supplied during the other crop growth stages. These levels are aligned with regional agricultural practices (Wu et al., 2009), similar to how maintaining soil moisture at 50%-60% of FC is generally sufficient in southern China, with 70% FC being slightly excessive (Xiao et al., 2011). The nitrogen application rates were designed with no nitrogen (N0, 0 kg ha^-1^), low nitrogen (N1, 130 kg ha^-1^), medium nitrogen (N2, 185 kg ha^-1^), and high nitrogen (N3, 240 kg ha^-1^), respectively. The specific experimental design was shown in **Table 1**. The urea (N≥46%) was selected as N fertilizer, with 60% applied as base fertilizer while the remained 40% applied accompanied with drip irrigation water during potato tuber formation. The calcium superphosphate fertilizer (P_2_O_5_≥12%, 150 kg ha^-1^) and the granular potassium fertilizer (K_2_O≥ 51%, 180kg ha^-1^) was supplied as P and K fertilizer, which were both used as basal fertilizers for potatoes. The water application amounts were monitored using water meters to maintain the designed soil water contents levels within the planned wet depth of 60 cm layer.

**Table 1:** Experimental design.

Treatments	Water deficit control					Nitrogen control	
	Soil moisture level	Seedling	Tuber formation	Tuber expansion	Starch accumulation	Nitrogen application level	Nitrogen application (kg ha^-1^)
W2N0	W_2_	45%~55%^a^	65%~75%	65%~75%	65%~75%	N_0_	0
W2N1		45%~55%	65%~75%	65%~75%	65%~75%	N_1_	130
W2N2		45%~55%	65%~75%	65%~75%	65%~75%	N_2_	185
W2N3		45%~55%	65%~75%	65%~75%	65%~75%	N_3_	240
W1N0	W_1_	55%~65%	65%~75%	65%~75%	65%~75%	N_0_	0
W1N1		55%~65%	65%~75%	65%~75%	65%~75%	N_1_	130
W1N2		55%~65%	65%~75%	65%~75%	65%~75%	N_2_	185
W1N3		55%~65%	65%~75%	65%~75%	65%~75%	N_3_	240
CK	W_0_	65%~75%	65%~75%	65%~75%	65%~75%	N_0_	0

a represents soil water moisture content within 60 cm layer (% of the field capacity). When the soil water moisture content was close to the lower limit, a certain amount of water was to be applied through drip irrigation to bring the soil moisture content maintained at the desined higher limit.

A soil auger was used to collect soil. The total sampling soil depth was 100 cm, with 20 cm increment for each layer. When the soil moisture within the planned wet layer of the experimental plot was lower than the designed lower limit, the crops were irrigated to bring the soil moisture to the design upper limit. This experiment used the main local potato variety “Atlantic”, which was a mid-to-late maturing variety. The plants were planted on April 26 and harvested on September 4, with a growth period of 132 days. The one-ridge-two rows drip irrigation planting mode was adopted with the ridge height of 40 cm, the ridge width of 70 cm, the row space of 40 cm, and the plant spacing of 15 cm, respectively. The planting density was 60, 636 ha^-1^, as shown in **Figures 1B, C**. Each treatment has three replications, with an area of 14.40 m^2^ (6.0 m×2.4 m) in each plot.

### 2.3 Measurements and calculations

#### 2.3.1 Plant growth

Three potatoes representing the overall growth of each experimental plot were selected, and the plant height, stem thickness, and dry matter were measured at seedling, tuber formation, tuber expansion, and starch accumulation.

Plant height and stem thickness were respectively measured using a roll ruler and vernier caliper with an accuracy of 0.01 cm and 0.01 mm, respectively.

The leaf area index (LAI) was measured by punching and weighing method:


(1) LAI=TLA / OLA

where TLA is the total leaf area of potato plants (m^2^), and OLA is the occupied land area (m^2^).

The dry matter was obtained by taking three complete plants from each plot, decomposed into roots, stems, leaves, and tubers. After cleaning the roots and stems, absorbing the surface moisture with blotting paper, the plants were dried at 105°C for 30 minutes in an oven until keeping a constant weight at 75°C, and the fresh weight and dry weight of each organ were weighed using a balance with an accuracy of 0.01 g.

#### 2.3.2 Nitrogen balance

The partial N mass balance in each treatment and check was calculated (Bohman et al., 2021b). In this method, the N inputs [N_Input_] were taken into account including initial soil inorganic N [N_initial soil_], N in seed tubers [N_seed_], fertilizer N applied [N_fertilizer_], N supplied by irrigation [N_irrigation_] and precipitation N [N_precipitation_], as well as estimated net soil N mineralization [N_mineralization_]. The N Outputs [N_Output_] were measured involving N [N_residual soil_] and measured plant N uptake [N_plant_]. The N_seed_ and N_precipitation_ were 11.98 kg N ha^-1^ and 7.2 kg N ha^-1^, and N_irrigation_ was W0 (19.31 kg N ha^-1^), W1, (18.00 kg N ha^-1^) and W2 (17.01 kg N ha^-1^) throughout the growing season for potatoes, respectively. The dry matter in each potato organ at each growth stage was crushed, sieved through a 0.5mm sieve, and digested with H_2_SO_4_-H_2_O_2_. The total nitrogen content of the plant was measured using the Kjeldahl method (K1160, China) (Sáez-Plaza et al., 2013). Soil $NO_{3^{-}}–N$ and NH_4_^+^-N contents within the 60 cm depth were measured using a spectrophotometer (T6 New Century, China) at 20 cm intervals from the sampling depth during potato harvest (Wang et al., 2018). The disparity difference between N input and output was defined as [N_unknow loss_] to quantify the unexplained nitrogen in the other component parts of the nitrogen balance.


(2) $$\begin{array}{c} {\text{N}}_{\text{Input }}={\text{N}}_{\text{initial soil}}+{\text{N}}_{\text{seed}}+{\text{N}}_{\text{precipitation}}+{\text{N}}_{\text{irrigation}}\\ \;\;\;+{\text{N}}_{\text{mineralization}}+{\text{N}}_{\text{fertilizer}} \end{array}$$

(3) $${\text{N}}_{\text{Output}}={\text{N}}_{\text{residual soil}}+{\text{N}}_{\text{plant}}$$

(4) $${\text{N}}_{\text{unknow loss}}={\text{N}}_{\text{Input}}+{\text{N}}_{\text{Output}}$$

#### 2.3.3 Potato yield

Ten potatoes were selected randomly in each experimental plot, and the average yield per plant was calculated by multiplying the planting density, which was used to determine the hectare yield of each plot.

#### 2.3.4 Irrigation water use efficiency

The irrigation water use efficiency (IWUE, kg m^-3^) and water use efficiency (WUE, kg m^-3^) were determined as follows:


(5) IWUE=Y/I

(6) WUE=Y/W

where Y is the total fresh tuber yield (kg ha^-1^), I is the amount of applied water (m^3^ ha^-1^), and W is the total amount of irrigation water and rainfall (m^3^ ha^-1^), representing total water input, respectively.

#### 2.3.5 Nitrogen use efficiency

The nitrogen uptake (N_uptake_, kg N ha^-1^) was determined as follows:


(7) $${\text{N}}_{\text{uptake}}=\sum_{\text{i}=1}^{\text{n}}{\text{m}}_{\text{i}}{\text{c}}_{\text{i}}$$

where the m_i_ is dry matter mass per organ (kg ha^-1^), and c_i_ is nitrogen content per organ (% N).

The soil *NO_3_^-^-N* content ( $NO_{3^{-}}–N$ , kg N ha^-1^) was calculated as following:


(8) $${\text{NO}}_{3}^{-}-\text{N}={\text{T}}_{\text{i}}\times {\text{BD}}_{\text{i}}\times \text{C}{\left({\text{NO}}_{3}^{-}-\text{N}\right)}_{\text{i}}$$

where i is the soil layer, BD_i_ is the soil bulk density, and C( $NO_{3^{-}}–N$ )_i_ is the soil $NO_{3^{-}}–N$ (mg kg^-1^) in the corresponding layer.

The soil NH_4_^+^-N content (NH_4_^+^-N, kg N ha^-1^) was calculated as following:


(9) $${\text{NH}}_{4}^{+}-\text{N}={\text{T}}_{\text{i}}\times {\text{BD}}_{\text{i}}\times \text{C}{\left({\text{NH}}_{4}^{+}-\text{N}\right)}_{\text{i}}$$

where C(NH_4_^+^-N)_i_ is the soil NH_4_^+^-N (mg kg^-1^) in the corresponding layer.

The nitrogen partial factor productivity (NPFP, kg kgN^-1^) was determined as following:


(10) $$\text{NPFP}=\text{Y}/{\text{N}}_{\text{fertilizer}}$$

where the N_fertilizer_ is the total N fertilizer application (kg N ha^-1^).

The crop nitrogen use efficiency (NUE_crop_) was calculated as following:


(11) $${\text{NUE}}_{\text{crop}}={\text{N}}_{\text{tuber}}/{\text{N}}_{\text{fertilizer}}$$

where the N_tuber_ is the tuber N accumulation (kg N ha^-1^).

The soil nitrogen use efficiency (NUE_soil_) was calculated as following:


(12) $${\text{NUE}}_{\text{soil}}={\text{N}}_{\text{plant}}/({\text{N}}_{\text{fertilizer}}+{\text{N}}_{\text{initial soil}})$$

where N_plant_ is the plant N accumulation (kg N ha^-1^), and the N_initial soil_ is the initial soil inorganic N content (kg N ha^-1^).

The systematic nitrogen use efficiency (SNUE) (Congreves et al., 2021) was calculated as following:


(13) $$\text{SNUE}={\text{N}}_{\text{tuber}}/({\text{N}}_{\text{tuber}}{\text{+N}}_{\text{unknow loss}})$$

where the N_unknow loss_ is the difference between nitrogen input and nitrogen output (kg N ha^-1^).

#### 2.3.6 Potato quality

At potato harvest, fresh samples of three potato tubers were taken from each plot to determine the starch, reducing sugar and vitamin C contents of the potato tubers. Iodine colorimetry was used to determine the starch content; 3,5-dinitrosalicylic acid colorimetry was used to determine the reducing sugar content; vitamin C content was determined by titration; and Kjeldahl nitrogen determination was used to determine the protein content of the tubers (Bogucka and Elżbieta, 2018).

### 2.4 Data processing and analysis

Data collation was performed using Microsoft Excel 2019 (Microsoft Corp, USA). The figures were graphed using Origin 2018 (Origin Lab Corp, USA). The statistical analyses were conducted using SPSS 27.0 (IBM Corp., USA). Specifically, the analysis of variance (ANOVA) was conducted using SPSS 27.0, and Duncan’s multiple comparisons method were used to evaluate the difference of significance (at a threshold of *p*<0.05).

## 3 Results

### 3.1 Plant growth

#### 3.1.1 Plant height

**Figure 3:**
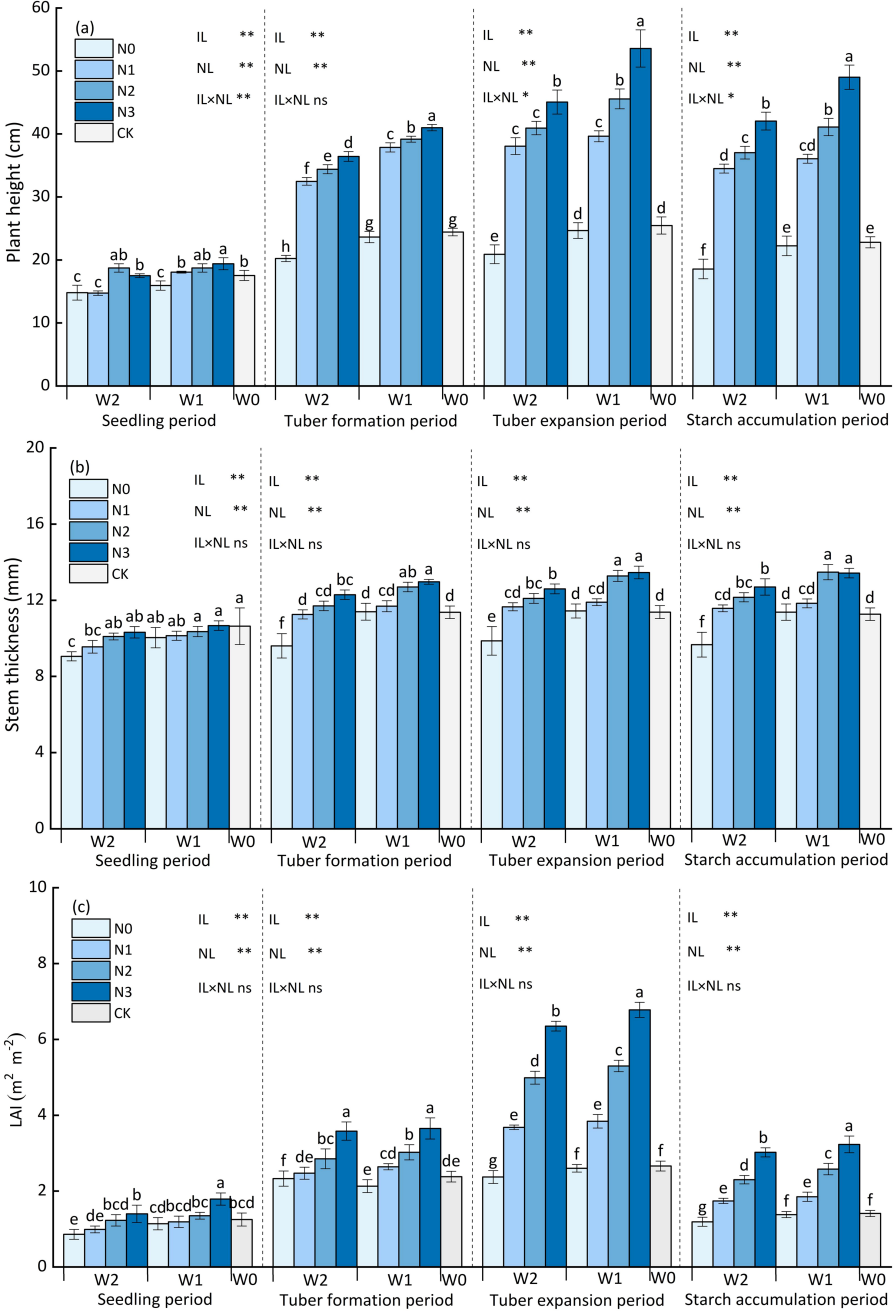
Effects of water and nitrogen regulation on potato growth. **(A)** represents the potato plant height, **(B)** represents the potato plant stem thickness, and **(C)** represents the potato Leaf Area Index. Bars represent the means ± standard deviation of the mean (*n*=3). Different letters above the bars indicate a significant difference at *p*<0.05 according to the Duncan test. *, ** and ns indicate the effect significance at the 0.05 level, the 0.01 level, and no significant effect, respectively.

The potato plant’s height, stem thickness, and LAI throughout the entirety of the growth period. All three indices exhibited an ascending trend, reaching their peak at the end of tuber expansion. The interaction between water and nitrogen had a significant effect on plant height (*p*<0.05). The single effect of nitrogen and water application on plant height had a highly significant effect (*p*<0.01) (**Figure 3A**). At the same irrigation level, the plant height increased with nitrogen application rate increasing. Among all the treatments and CK, the plant height in W1N3 was recorded the highest, significantly enhanced by 17.56% to 156.32% compared with the other treatments. At the W1 irrigation level, the plant height in W1N3 treatment was 17.56%, 35.18%, 117.15%, and 110.33% significantly higher than W1N2, W1N1, W1N0 and CK treatment. At the W2 irrigation level, the plant height in W2N3 treatment was 10.11%, 18.39%, 115.65%, and 82.69% higher than W2N2, W2N1, W2N0 and CK treatment. Likewise, at the same nitrogen application level, the plant height increased with the irrigation amount increasing. At the N2, and N3 application levels, the plant heigh of W1 treatment was 18.04%, and 18.60% significantly higher than W2 treatment. In addition, at the N0 application level, the plant height of W1N0 treatment was significantly higher than W2N0 treatment by 18.04%, while there was no significant difference (*p*>0.05) in the plant heights between the W1N0 and CK treatment, indicating that potato plant height had certain compensatory growth effect after rehydration when experiencing a mild water deficit at the seedling.

#### 3.1.2 Stem thickness

As shown in **Figure 3B**, the interaction between water and nitrogen application had no significant effect on potato stem thickness *(p*>0.05). The single effect of nitrogen and water application on stem thickness had a highly significant effect (*p*<0.01). At the same irrigation level, the stem thickness increased with the nitrogen application rate increasing, but except for N2 and N3 nitrogen application level. Among all the treatments and CK, the stem thickness in W1N3 was recorded the highest, significantly enhanced by 6.39% to 36.37% compared with the other treatments. Equally, at the same nitrogen application level, the stem thickness increased with irrigation amount increasing. At the N2, and N3 application levels, the stem thickness of W1 treatment was 11.36%, and 6.39% significantly higher than W2 treatment. Furthermore, at the N0 application level, the stem thickness of W1N0 treatment was significantly higher than W2N0 treatment by 5.46%, while there was no significant difference (*p*>0.05) in the stem thickness between the W1N0 and CK treatment, indicating that potato stem thickness also had certain compensatory growth effect after rehydration when experiencing a mild water deficit at the seedling.

#### 3.1.3 Leaf area index

As shown in **Figure 3C**, the interaction between water and nitrogen application had no significant effect on potato LAI (*p*>0.05). The single effect of nitrogen and water application on LAI had a highly significant effect (*p*<0.01). At the same irrigation level, LAI increased with nitrogen application rate increasing. At the W1 irrigation level, the LAI value of W1N3 treatment was highest, which was greater by 27.92%, 76.56%, and 160.77% compared to W1N2, W1N1, and W1N0 treatment. At the W2 irrigation level, the LAI value of W2N3 treatment was 27.05%, 72.28%, and 167.51% significantly higher than W2N2, W2N1, and W2N0. Except for N0 and N1 application level. Likewise, at the same nitrogen application level, the LAI increased with irrigation amount increasing. At the N2, and N3 application levels, the LAI value of W1 treatment was 6.21%, and 6.90% significantly higher than W2 treatment. Same as above, at the N0 application level, the LAI value of W1N0 treatment was significantly higher than W2N0 treatment by 9.70%, while there was no significant difference (*p*>0.05) in the LAI between the W1N0 and CK treatment, indicating that LAI also had certain compensatory growth effect after rehydration when experiencing a mild water deficit at the seedling.

#### 3.1.4 Dry matter accumulation

**Figure 4:**
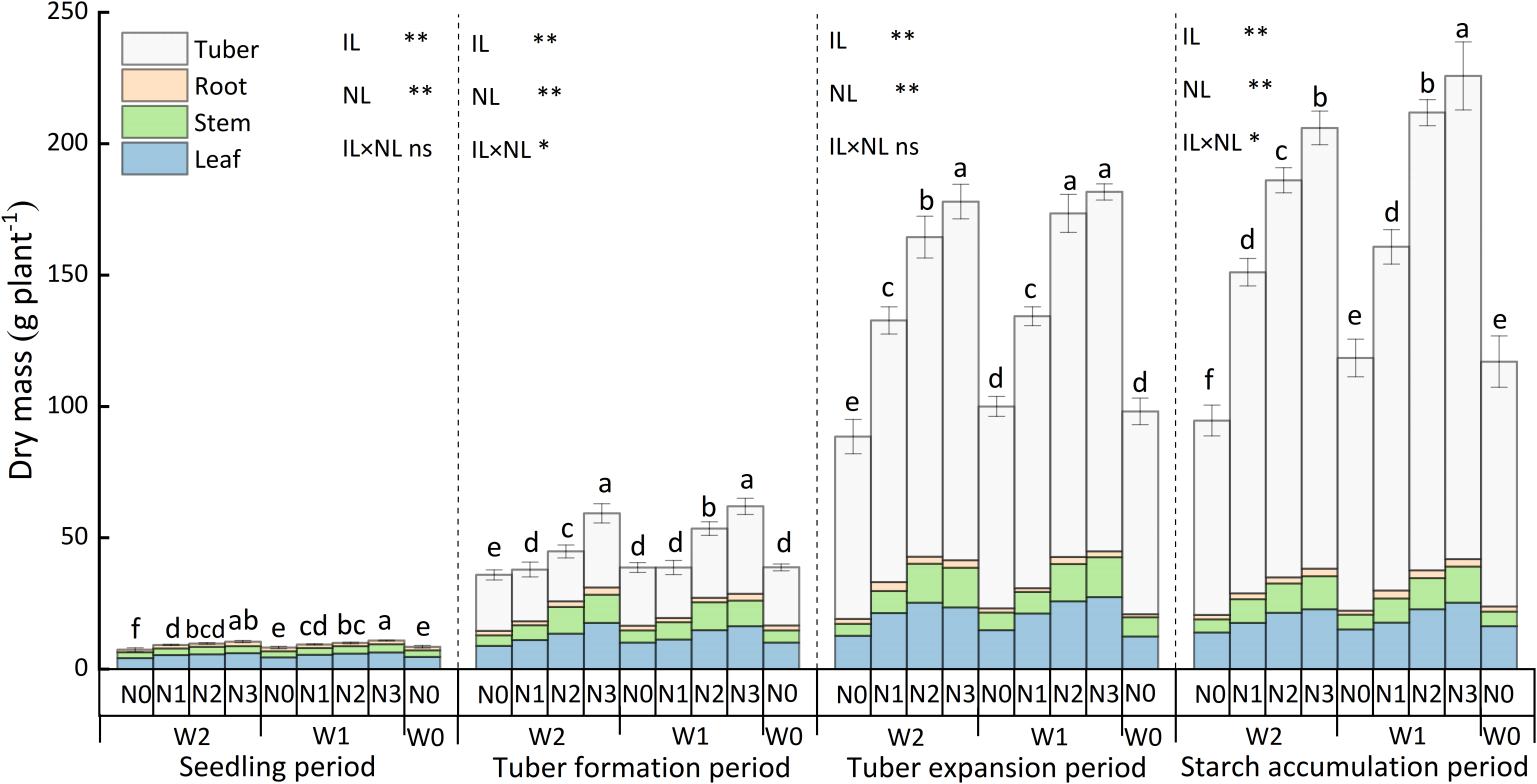
The dry matter accumulation of potatoes during the entire growth period. Bars represent the means ± standard deviation of the mean (*n*=3). Different letters above the bars indicate a significant difference at *p*<0.05 according to the Duncan test. *, ** and ns indicate the effect significance at the 0.05 level, the 0.01 level, and no significant effect, respectively.

The water and nitrogen application had a highly significant effect (*p*<0.01) on the dry matter accumulation of potato during the entire growth period, and the interaction between water and nitrogen also had a significant effect (*p*<0.05) on the dry matter accumulation at tuber formation and starch accumulation (**Figure 4**). The dry matter accumulation in different potato tissues at various growth stages was in the order of tuber > leaf > stem > root except for that at seedling. At the same irrigation level, the dry matter accumulation increased with the nitrogen application rate increasing. During the starch accumulation period. At the W1 irrigation level, the dry matter accumulation of W1N3 treatment was highest, which was greater by 6.47%, 40.47%, 130.67% and 93.34% compared to W1N2, W1N1, W1N0, and CK treatment. At the W2 irrigation level, the LAI value of W2N3 treatment was 10.73%, 36.46%, 118.55%, and 76.40% significantly higher than W2N2, W2N1, W2N0, and CK treatment. At the same nitrogen application level, the dry matter accumulation increased with the irrigation amount increasing. During the starch accumulation period. At the N2, and N3 application levels, the dry matter accumulation of W1 treatment was 14.00%, and 9.61% significantly higher than W2 treatment, but there was no significant difference (*p*>0.05) in the dry matter accumulation between W1N1 and W2N1, and between W1N0 and W2N0 treatments.

### 3.2 Plant nitrogen

**Figure 5:**
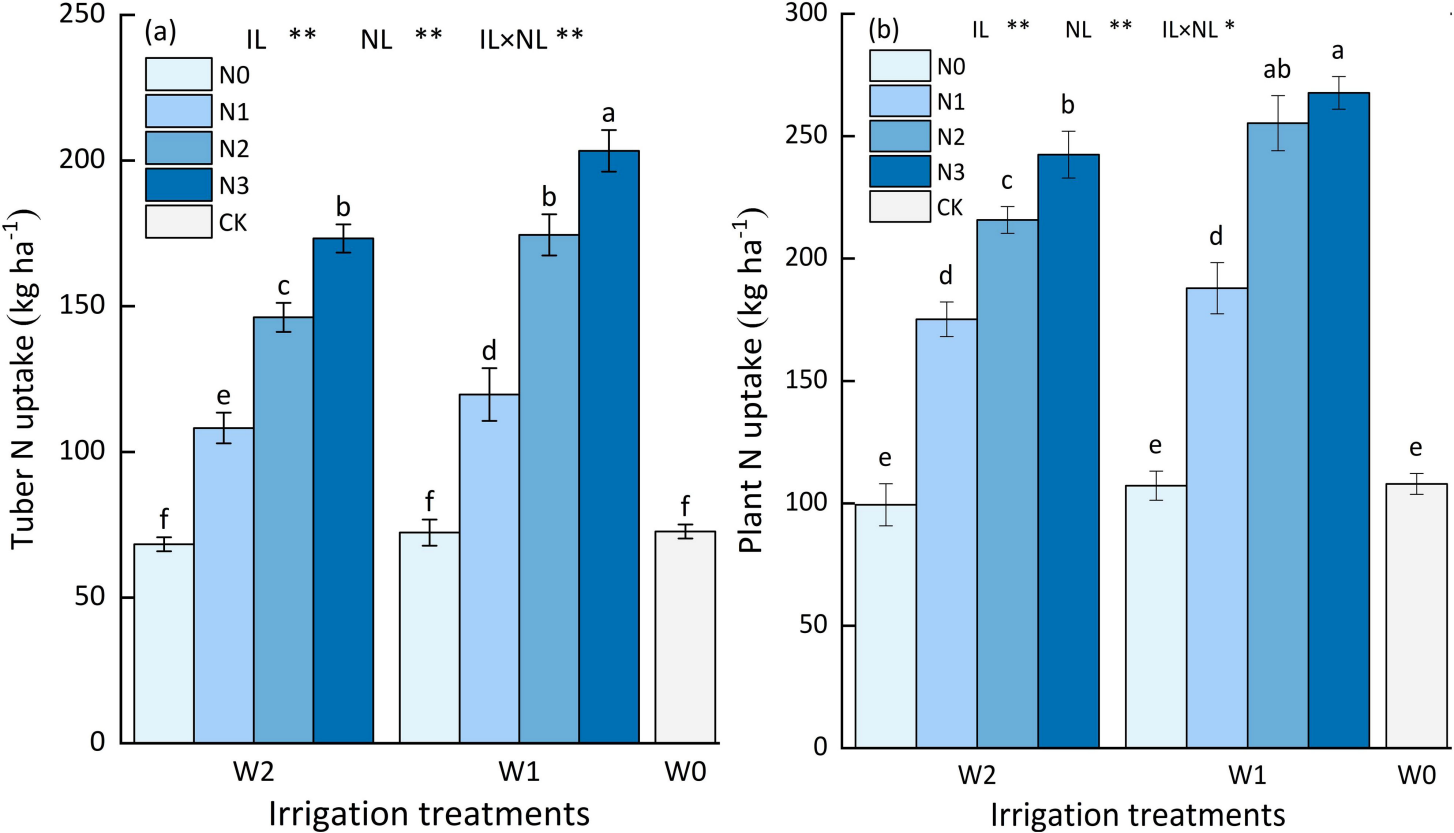
The impact of different water and nitrogen treatments on the N uptake. **(A)** The impact of different water-nitrogen treatments on the tuber N uptake. **(B)** The impact of different water-nitrogen treatments on the plant N uptake. Bars represent the means ± standard deviation of the mean (*n*=3). Different letters above the bars indicate a significant difference at *p*<0.05 according to the Duncan test. *, ** and ns indicate the effect significance at the 0.05 level, the 0.01 level, and no significant effect, respectively.

The interplay between water and nitrogen application, and the single effect of nitrogen and water application on tuber N uptake were highly significant (*p*<0.01), as shown in **Figure 5A**. At the same irrigation level, the tuber N uptake increased with nitrogen application rate increasing. Among all the treatments and CK, the tuber N uptake in W1N3 was recorded the highest, significantly improved by 17.56% to 156.32% compared with the other treatments. At the W1 irrigation level, the tuber N uptake of W1N3 treatment was 16.27%, 70.14%, 180.15%, and 180.88% significantly higher than W1N2, W1N1, W1N0, and CK treatment. At the W2 irrigation level, the tuber N uptake of W2N3 treatment was 18.52%, 59.83%, 151.37%, and 139.19%, significantly higher than W2N2, W2N1, W2N0, and CK treatment. Likewise, at the same nitrogen application level, the tuber N uptake increased with water application increasing, except for N0 application level. At the N1, N2, and N3 application levels, the tuber N uptake of W1 treatment was 10.31%, 19.62%, and 17.43% significantly higher than W2 treatment, but there was no significant difference (*p*>0.05) the tuber N uptake among W1N0, W2N0, and CK treatment.

The interaction between water and nitrogen application had a significant effect on plant N uptake (*p*<0.05). The single effect of nitrogen and water application on plant N uptake were highly significant (*p*<0.01), as shown in **Figure 5B**. At the same irrigation level, the plant N uptake increased with nitrogen application rate increasing. Among all the treatments and CK, the plant N uptake in W1N3 was recorded the highest, significantly improved by 4.83% to 169.32% compared with the other treatments. At the W1 irrigation level, the plant N uptake of W1N3 treatment was 4.83%, 42.46%, 149.76%, and 147.70% higher than W1N2, W1N1, W1N0, and CK treatment. At the W2 irrigation level, the plant N uptake of W2N3 treatment was 12.36%, 38.39%, 143.84%, and 124.60%, higher than W2N2, W2N1, W2N0, and CK treatment. Likewise, at the same nitrogen application level, the tuber N uptake increased with water application increasing, except for N0 application level. At the N1, N2, and N3 application levels, the tuber N uptake of W1 treatment was 10.31%, 19.62%, and 17.43% significantly higher than W2 treatment. In addition, at the N0 application level, the plant N uptake among the W0N0, W1N0, and W2N0 treatments were 107.94 kg N ha^-1^, 107.17 kg N ha^-1^, and 99.42 kg N ha^-1^, respectively, indicating that the average value of INS is 104.84 kg N ha^-1^.

### 3.3 Soil $NO_{3^{-}}–N$ and NH_4_^+^-N

#### 3.3.1 Soil $NO_{3^{-}}–N$

**Figure 6:**
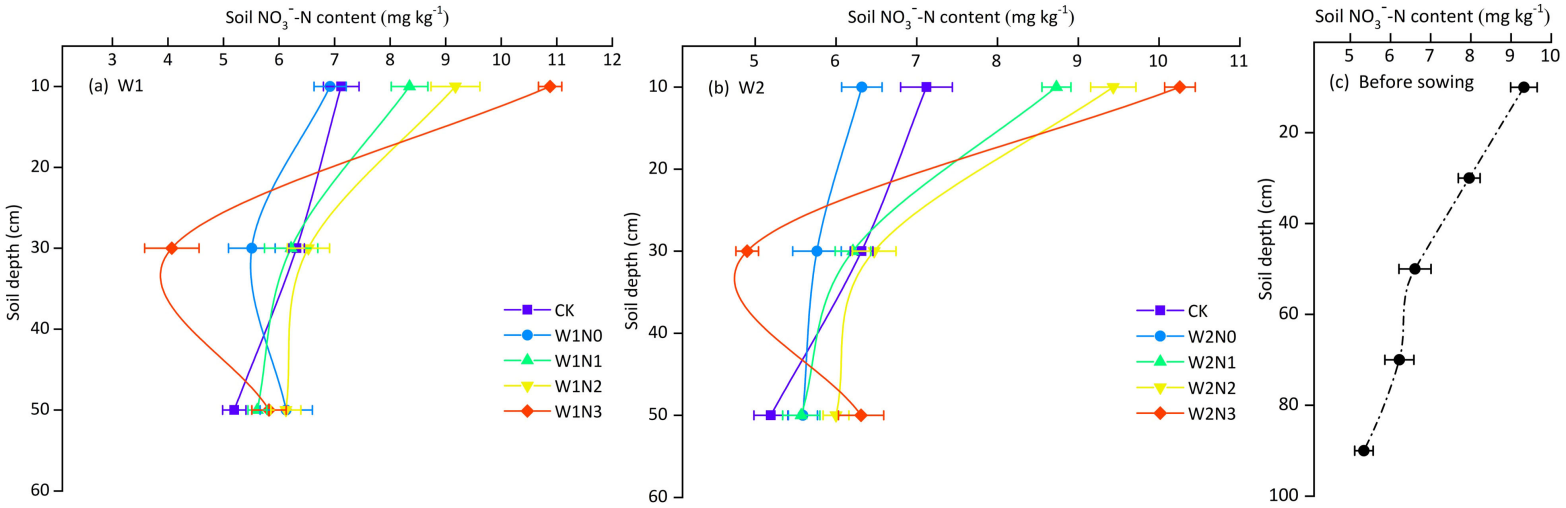
The distribution of *NO_3_^-^-N* in different soil depth. **(A, B)** The distribution of *NO_3_^-^-N* with soil depth under W1 and W2 irrigation level. **(C)** The distribution of *NO_3_^-^-N* with soil depth before sowing. Bars represent the means ± standard deviation of the mean (*n*=3).

In potato fields, the soil $NO_{3^{-}}–N$ content declined followed by an increase with soil depth at harvest period (**Figures 6A–C**). The 0-20 cm soil layer in each treatment displayed the highest $NO_{3^{-}}–N$ content, ranging from 6.32 to 10.88 mg kg^-1^, and treatments applying N significantly increased $NO_{3^{-}}–N$ content in this soil layers compared with the N0 treatment and CK. Within the 20-40 cm soil layer, the soil $NO_{3^{-}}–N$ content was significantly lower under the N3 application level than under the other N application treatments. Except for the N3 application level, the lowest $NO_{3^{-}}–N$ content was observed in the 40-60 cm layer of each treatment, ranging from 5.19 to 6.31 mg kg^-1^. Compared with the initial $NO_{3^{-}}–N$ content in the soil before sowing: (1) In the 0-20 cm soil layer, the N3 application level significantly increased the $NO_{3^{-}}–N$ content, the $NO_{3^{-}}–N$ content of N2 application level was basically stable, the $NO_{3^{-}}–N$ content of N1 application level was slightly reduced, and the N0 application level significantly reduced the $NO_{3^{-}}–N$ content; (2) In the 20-40 cm soil layer, the $NO_{3^{-}}–N$ content of all treatments was reduced, in which the N3 treatment showed the largest decrease in the $NO_{3^{-}}–N$ content; (3) In the 40-60 cm soil layer, the $NO_{3^{-}}–N$ content decreased from 4.75% to 27.36%.

#### 3.3.2 Soil NH_4_^+^-N

**Figure 7:**
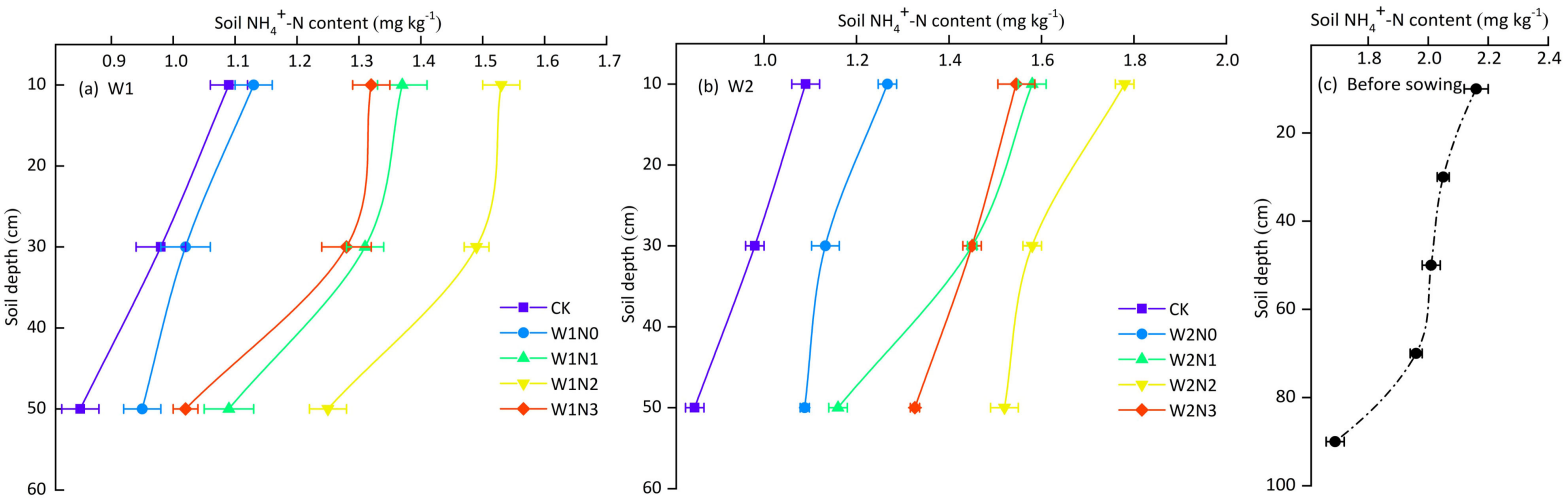
The distribution of NH_4_^+^-N in different soil depth. **(A, B)** The distribution of NH_4_^+^-N with soil depth under W1 and W2 irrigation level. **(C)** The distribution of NH_4_^+^-N with soil depth before sowing. Bars represent the means ± standard deviation of the mean (*n*=3).

In the potato field, the soil NH_4_^+^-N content showed a decreasing trend with the soil depth increasing at harvest (**Figures 7A–C**). Among the treatments and CK, the highest NH_4_^+^-N content was found in the 0-20 cm soil layer, ranging from 1.09 to 1.79 mg kg^-1^, and treatments applying N significantly increased NH_4_^+^-N content compared with N0 treatment and CK; In the 20-40 cm soil layer, the soil NH_4_^+^-N content of the N0 application level was significantly lower than that of the other N application treatments; In the 40-60 cm soil layer, the NH_4_^+^-N content was the lowest among the treatments, ranging from 0.85 to 1.52 mg kg^-1^. Compared with the initial NH_4_^+^-N content before sowing, in the soil layers of 0-20 cm, 20-40 cm, and 40-60 cm, the NH_4_^+^-N content was reduced in all treatments, with the smallest decreases in the N2 application level, ranging from 17.59% to 24.38%; the NH_4_^+^-N content of the N0 and CK treatments showed the largest decrease, ranging from 48.96% to 57.71%.

### 3.4 Nitrogen balance

**Figure 8:**
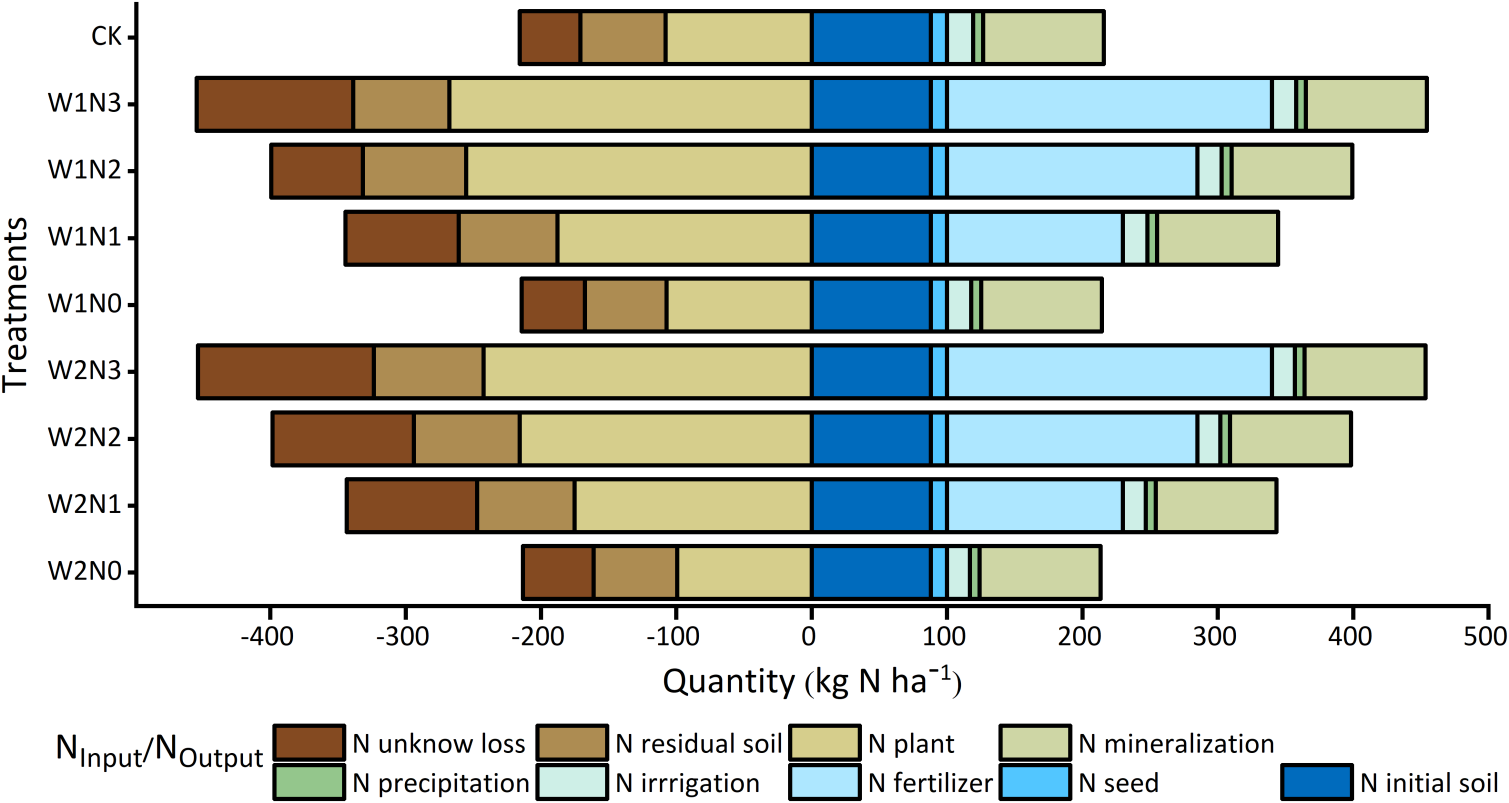
Components of N_Input_ and N_Output_ shown for different water and nitrogen strategy.

The primary sources of N_Input_ in potato fields are N_fertilizer_ and N_mineralization_ (**Figure 8**). Variations in N_Input_ among different treatments can be primarily attributed to differences in N_fertilizer_. In this specific study, the amount of soil N_mineralization_ was measured to be 89.33 kg ha^-1^ under the drip irrigation water nitrogen regulation mode. Additionally, N_seed_ and N_precipitation_ accounted for 11.98 and 7.2 kg ha^-1^, respectively. The differences in N_irrigation_ among the various irrigation treatments were found to be minimal, with values of 17.01, 18.00, and 19.42 kg ha^-1^ for W0, W1, and W2, respectively. However, the major N_Output_ in potato fields were determined to be N_plant_ and N_unknown loss_. N_plant_ contributed to a range of 46.58% to 63.93% of the total N_Output_, with the W1N2 treatment exhibiting the highest percentage of N_Output_. N_unknown_ loss accounted for 17.00% to 28.65% of the primary N_Output_ component, with the W1N2 treatment demonstrating the lowest N_unknown loss_ of 67.89 kg ha^-1^. The N3 treatment exhibited the highest N_unknown_ loss under the same irrigation level. Furthermore, at the same N application level, the W1 treatment displayed lower N_unknown loss_ compared to the W0 treatment.

### 3.5 Potato yield, irrigation water use efficiency, and nitrogen use efficiency

#### 3.5.1 Yield

The interaction between water and nitrogen had a significant effect on the potato yield (*p*<0.05). The single effect of nitrogen application on the potato yield were highly significant (*p*<0.01), as shown in **Table 2**. At the same irrigation level, the potato yield increased with nitrogen application rate increasing. Among all the treatments and CK, the potato yield of W1N3 was recorded the highest (45.37 t ha^-1^), significantly improved by 5.12% to 91.29% compared with the other treatments. At the W1 irrigation level, the potato yield of W1N3 treatment was 5.11%, 43.33%, and 75.12% higher than W1N2, W1N1, and W1N0 treatment, but there was no statistically significant difference between W1N3 and W1N2 treatments. At the W2 irrigation level, the potato yield of W2N3 treatment was 9.33%, 38.10%, and 77.16% significantly higher than W2N2, W2N1, and W2N0 treatments. The single effect of water on potato yield was also significant (*p*<0.01), except for N0 and N1 application level. At the same nitrogen application level, the potato yield increased with irrigation amount increasing. At the N2, and N3 application levels, the potato yield of W1 treatment was 12.31%, and 7.97% significantly higher than W2 treatment, but there was no significant difference (*p*>0.05) in the potato yield between N1 and N0 nitrogen application level.

**Table 2:** Potato yield, water and nitrogen use efficiency in potato field under different water-nitrogen regulation strategies.

Treatments	Y (t ha^-1^)	IWUE (kg m^-3^)	NUE indices			
			Fertilizer-based NPFP (kg kgN^-1^)	Plant-based NUE_crop_	Soil-based NUE_soil_	System-based SNUE
W2N0	23.72 ± 1.86(e)	8.93 ± 0.96(f)	–	–	1.13 ± 0.05 (b)	0.57 ± 0.01 (e)
W2N1	30.42 ± 1.75 (d)	11.73 ± 0.72 (d)	234.03 ± 14.26 (a)	0.83 ± 0.04 (c)	0.80 ± 0.03 (de)	0.53 ± 0.01 (f)
W2N2	38.43 ± 1.75(c)	16.93 ± 0.77 (c)	207.74 ± 9.47 (b)	0.79 ± 0.03 (d)	0.79 ± 0.02 (de)	0.58 ± 0.01 (e)
W2N3	42.02 ± 1.36 (b)	19.62 ± 0.63 (ab)	175.07 ± 5.66 (c)	0.72 ± 0.02 (d)	0.74 ± 0.03 (e)	0.57 ± 0.01 (e)
W1N0	25.91 ± 0.65 (e)	10.25 ± 0.26(e)	–	–	1.22 ± 0.07 (a)	0.61 ± 0.01 (cd)
W1N1	31.65 ± 1.60 (d)	12.63 ± 0.64 (d)	243.47 ± 12.27 (a)	0.92 ± 0.07 (ab)	0.86 ± 0.05 (cd)	0.59 ± 0.02 (de)
W1N2	43.16 ± 1.44 (ab)	20.51 ± 0.68 (a)	233.28 ± 7.76(a)	0.95 ± 0.04 (a)	0.94 ± 0.04 (c)	0.72 ± 0.01 (a)
W1N3	45.37 ± 1.45 (a)	19.26 ± 0.61(b)	189.03 ± 6.04(c)	0.85 ± 0.03 (bc)	0.82 ± 0.02 (de)	0.64 ± 0.01 (b)
CK	26.26 ± 1.38 (e)	9.72 ± 0.51 (ef)	–	–	1.23 ± 0.05 (a)	0.62 ± 0.01 (c)
Significance levels						
IL	**	**	**	**	**	**
NL	**	**	**	**	**	**
IL×NL	*	**	ns	ns	ns	**

Y indicates potato tuber yield, IWUE indicates irrigation water use efficiency, NPFP indicates nitrogen partial factor productivity, NUEcrop indicates crop-based nitrogen use efficiency, NUEsoil indicates soil-based nitrogen use efficiency, and SNUE indicates system-based nitrogen use efficiency. Different letters indicate a significant difference at *p*<0.05 according to the Duncan test. *, ** and ns indicate the effect significance at the 0.05 level, the 0.01 level, and no significant effect, respectively.

#### 3.5.2 Irrigation water use efficiency

The interplay between water and nitrogen application, and the single effect of nitrogen and water application on IWUE were highly significant (*p*<0.01), as shown in **Table 2**. At the same irrigation level, the IWUE increased with nitrogen application rate increasing. Among all the treatments and CK, the IWUE of W1N2 was recorded the highest, significantly improved by 4.54% to 110.96% compared with the other treatments. At the W1 irrigation level, the IWUE of W1N2 treatment was 6.56%, 62.43%, 100.14%, and 110.96% significantly higher than W1N3, W1N1, W1N0, and CK treatment. At the W2 irrigation level, the IWUE of W2N3 treatment was 15.91%, 67.27%, 119.79%, and 101.80% significantly higher than W2N2, W2N1, W2N0, and CK treatment. Likewise, at the N2 and N0 application levels, the IWUE increased with water application increasing, and the IWUE of W1 irrigation was 21.17% and 14.80% significantly higher than W2 treatment.

#### 3.5.3 Nitrogen use efficiency

The single effect of nitrogen and water application had highly significant effects on fertilizer-based NPFP, plant-based NUE_crop_, and soil-based NUE_soil_ (*p*<0.01). NPFP significantly decreases with nitrogen application rate increasing. Among them, the NPFP of W1N1 treatment is the highest (243.47 kg kgN^-1^), followed by W1N2 (233.28 kg kgN^-1^), and there is no statistical difference between the two (*p*>0.05). The NUE_crop_ at W1 level is significantly higher than that at W0, and the NUE_crop_ decreases with nitrogen application rate increasing, except for W1N2, of which, the NUE_crop_ of W1N2 treatment is the highest (0.95). The interplay between water and nitrogen application, and the single effect of nitrogen and water application on system-based SNUE were highly significant (*p*<0.01). Among all the treatments and CK, the SNUE of W1N2 was recorded the highest (0.72), significantly improved by 12.50% to 35.85% compared with the other treatments. At the same irrigation level, SNUE was highest under N2 application level. At the N3, N2, N1 and N0 application levels, the SNUE of W1 treatment was 12.28%, 24.14%, 11.32% and 7.97% significantly higher than W2 treatment.

### 3.6 Potato quality

#### 3.6.1 Starch content

**Figure 9:**
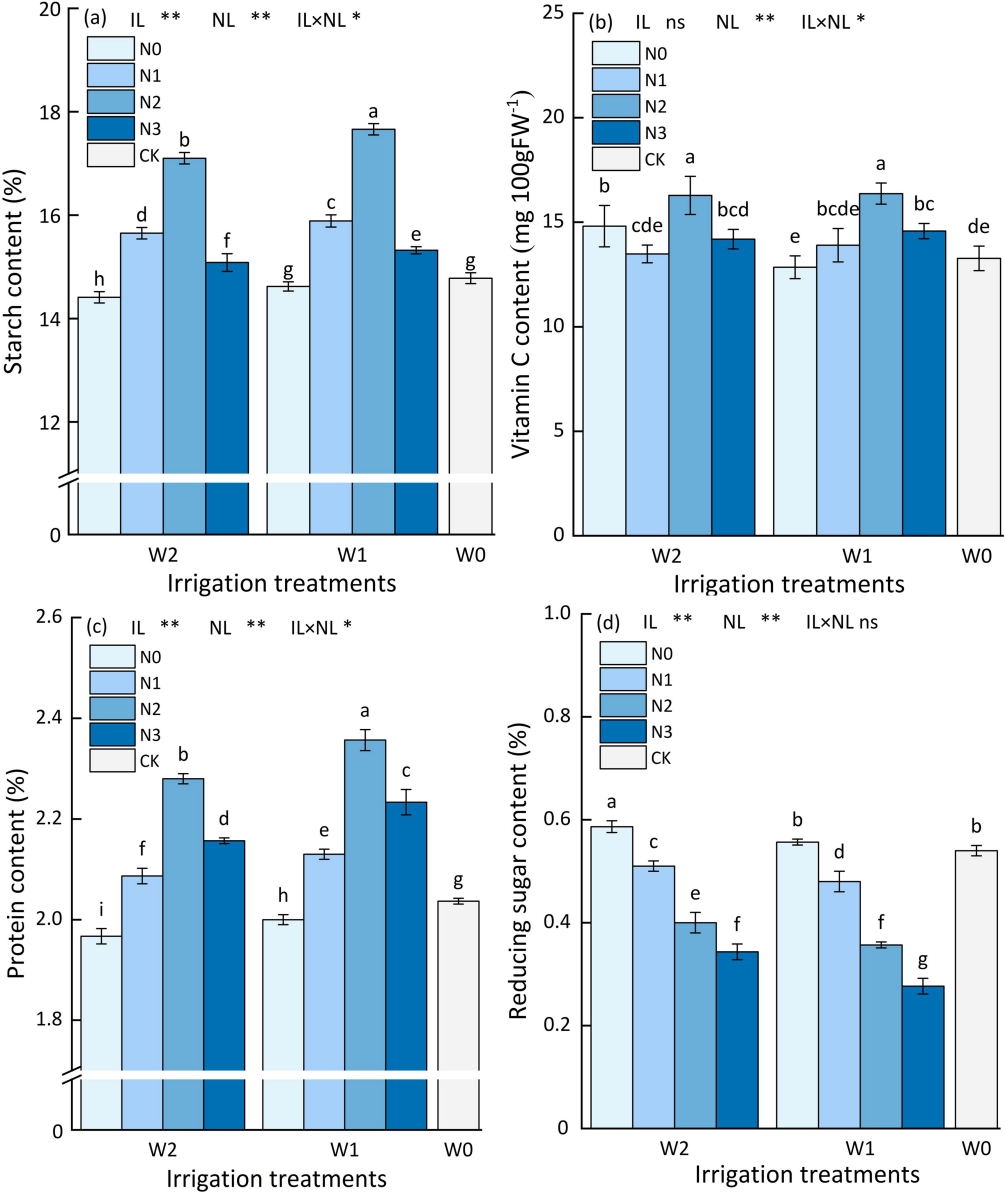
Different water and nitrogen regulated potato quality (Fresh base %). **(A–D)** Describes changes in starch, vitamin c, protein, and reducing sugar content. Bars represent the means ± standard deviation of the mean (*n*=3). Different letters above the bars indicate a significant difference at *p*<0.05 according to the Duncan test. *, ** and ns indicate the effect significance at the 0.05 level, the 0.01 level, and no significant effect, respectively.

The interplay between water and nitrogen application on potato starch content had a significantly influences (*p*<0.05). The single effect of nitrogen and water application on potato starch content were highly significant (*p*<0.01), as shown in **Figure 9A**. The highest potato starch content was found in W1N2 treatment (17.66%), followed by W2N2 treatment (17.10%), the lowest in W2N0 treatment (14.41%), and the starch content of CK treatment was 14.78%. Under the same irrigation level, the potato starch content showed an increasing and then decreasing trend with the increase of nitrogen application, and the highest starch content was found at the N2 level, in which the potato starch content of the W1N2 treatment was higher than that of the W1N3, W1N1, W1N0 and CK treatments by 15.27%, 11.14%, 20.79% and 19.49%, respectively. The potato starch content of W2N2 treatment was higher than that of W2N3, W2N1, W2N0 and CK treatments by 13.40%, 9.27%, 18.67% and 15.70%, respectively. Under the same level of nitrogen application, the potato starch content increased with the increase of irrigation, in which the starch content of W1N3 increased by 3.27% compared with that of W2N3 treatment, and the starch content of CK increased by 2.57% compared with that of W2N0 treatment, but there was no significant difference in the starch content of CK and W1N0 treatments.

#### 3.6.2 Vitamin C content

The single effect of nitrogen and water application on potato Vitamin C content were highly significant (*p*<0.01), as shown in **Figure 9B**. The highest Vitamin C content was found in W1N2 treatment (16.37 mg 100 g FW^-1^), followed by W2N2 treatment (16.28 mg 100 g FW^-1^), the lowest in W1N0 treatment (12.84 mg 100 g FW^-1^), and the Vitamin C content of CK treatment was 13.27 mg 100 g FW^-1^. Under the same irrigation level, the potato Vitamin C content showed an increasing and then decreasing trend with the increase of nitrogen application, and the highest Vitamin C content was found at the N2 level, in which the potato Vitamin C content of the W1N2 treatment was higher than that of the W1N3, W1N1, W1N0 and CK treatments by 12.28%, 17.77%, 27.49% and 23.36%, respectively. The potato Vitamin C content of W2N2 treatment was higher than that of W2N3, W2N1, W2N0 and CK treatments by 14.73%, 20.77%, 9.93% and 22.68%, respectively. Under the same level of nitrogen application, the potato Vitamin C content increased with the increase of irrigation, in which the Vitamin C content of CK increased by 3.12% compared with that of W1N0 treatment, but the Vitamin C content of CK decreased by 10.40% compared with that of W2N0 treatment.

#### 3.6.3 Protein content

The interplay between water and nitrogen application on potato protein content had a significantly influences (*p*<0.05). The single effect of nitrogen and water application on potato protein content were highly significant (*p*<0.01), as shown in **Figure 9C**. The highest potato protein content was found in W1N2 treatment (2.36%), followed by W2N2 treatment (2.28%), the lowest in W2N0 treatment (1.97%), and the protein content of CK treatment was 2.04%. Under the same irrigation level, the potato protein content showed an increasing and then decreasing trend with the increase of nitrogen application, and the highest protein content was found at the N2 level, in which the potato protein content of the W1N2 treatment was higher than that of the W1N3, W1N1, W1N0 and CK treatments by 5.83%, 10.80%, 18.00% and 15.69%, respectively. The potato protein content of W2N2 treatment was higher than that of W2N3, W2N1, W2N0 and CK treatments by 8.00%, 9.09%, 15.74% and 11.76%, respectively. Under the same level of nitrogen application, the potato protein content increased with the increase of irrigation, in which the protein content of CK compared with that of W1N3 and W2N3 treatment increased by 2.00% and 3.55%, respectively.

#### 3.6.4 Reduced sugar content

The single effect of nitrogen and water application on potato Reduced sugar content were highly significant (*p*<0.01), as shown in **Figure 9D**. The highest potato Reduced sugar content was found in W2N0 treatment (0.59%), followed by W1N0 treatment (0.56%), the lowest in W1N3 treatment (0.28%), and the Reduced sugar content of CK treatment was 0.54%. Under the same irrigation level, the potato Reduced sugar content showed a decreasing trend with the increase of nitrogen application, and the highest Reduced sugar content was found at the N0 level, in which the potato Reduced sugar content of the W1N0 treatment was higher than that of the W1N1, W1N2, W1N3 and CK treatments by 15.69%, 47.50%, 73.43% and 9.26%, respectively. The potato Reduced sugar content of W2N0 treatment was higher than that of W2N1, W2N2, W2N3 and CK treatments by 16.67%, 55.56%, 50.00% and 3.70%, respectively. Under the same level of nitrogen application, the potato Reduced sugar content increased with the increase of irrigation, in which the Reduced sugar content of W0N2 increased by 21.43% compared with that of W1N3 treatment, and the Reduced sugar content of CK decreased by 5.36% compared with that of W2N0 treatment, but there was no significant difference in the Reduced sugar content of CK and W1N0 treatments.

### 3.7 Optimization of water and nitrogen regulation strategies for potatoes

#### 3.7.1 Selection of comprehensive reference factors for water and nitrogen regulation

In this study, the potato economic efficiency index - potato yield (Y, t ha^-1^), the main quality index - starch content (SC, %), water use efficiency (WUE, kg m^-3^), as well as nitrogen use efficiency and environmental effect index - system nitrogen use efficiency (SNUE) were selected as the comprehensive performance reference factors in the study of water and nitrogen regulation in drip irrigation potatoes under the membrane, and the comprehensive performance reference factors were constructed, shown in **Table 3**.

**Table 3:** Experimental data of comprehensive performance reference factors of optimal water and nitrogen regulation strategy for potato under membrane drip irrigation.

Treatment	Y (t ha^-1^)	SC (%)	WUE (kg m^-3^)	SNUE
W2N0	23.71	14.41	7.46	0.56
W2N1	30.43	15.65	9.4	0.53
W2N2	38.44	17.10	11.26	0.58
W2N3	42.02	15.08	11.94	0.56
W1N0	25.9	14.62	8.09	0.62
W1N1	31.66	15.89	9.32	0.58
W1N2	43.16	17.66	12.36	0.72
W1N3	45.37	15.32	11.88	0.63
CK	26.26	14.78	7.57	0.61

Y indicates potato tuber yield, SC indicates the starch content of potato tubers, WUE indicates water use efficiency, and SNUE indicates system-based nitrogen use efficiency.

#### 3.7.2 Constructing a one-factor judgment matrix

Potato yield, starch content, WUE and SNUE four comprehensive performance reference factors to establish a single-factor judgment matrix, take the maximum value of each factor as 1, through the calculation of other treatments and the maximum value of the factor ratio, and then construct a single factor judgment matrix for:


$$\begin{array}{c} {\text{M}}_{4\times 9}=[\begin{array}{c} \text{Yield}\;(\text{Y})\\ \text{Starch content}\;(\text{SC})\\ \text{Water use efficiency}\;(\text{WUE})\\ \text{Systematic nitrogen use efficiency}\;(\text{SNUE}) \end{array}]\\ =\left[\begin{array}{ccccccccc} \text{W}2\text{N}0 & \text{W}2\text{N}1 & \text{W}2\text{N}2 & \text{W}2\text{N}3 & \text{W}1\text{N}0 & \text{W}1\text{N}1 & \text{W}1\text{N}2 & \text{W}1\text{N}3 & \text{CK}\\ 23.72 & 30.43 & 38.44 & 42.02 & 25.90 & 31.66 & 43.16 & 45.37 & 26.26\\ 14.41 & 15.65 & 17.10 & 15.08 & 14.62 & 15.89 & 17.66 & 15.32 & 14.78\\ 7.46 & 9.40 & 11.26 & 11.94 & 8.09 & 9.32 & 12.36 & 11.88 & 7.57\\ 0.56 & 0.53 & 0.58 & 0.56 & 0.62 & 0.58 & 0.72 & 0.63 & 0.61 \end{array}\right]\\ =\left[\begin{array}{ccccccccc} \text{W}2\text{N}0 & \text{W}2\text{N}1 & \text{W}2\text{N}2 & \text{W}2\text{N}3 & \text{W}1\text{N}0 & \text{W}1\text{N}1 & \text{W}1\text{N}2 & \text{W}1\text{N}3 & \text{CK}\\ 0.523 & 0.671 & 38.44 & 42.02 & 25.90 & 31.66 & 43.16 & 45.37 & 26.26\\ 0.816 & 0.886 & 17.10 & 15.08 & 14.62 & 15.89 & 17.66 & 15.35 & 14.78\\ 0.604 & 0.761 & 11.26 & 11.94 & 8.09 & 9.32 & 12.36 & 11.88 & 7.57\\ 0.778 & 0.736 & 0.58 & 0.56 & 0.62 & 0.58 & 0.72 & 0.63 & 0.61 \end{array}\right] \end{array}$$

#### 3.7.3 Construction of the matrix of weight coefficients for the individual participating factors

In this study, the size of the weighting coefficients of the four comprehensive reference factors, namely potato yield, starch content, WUE and SNUE, was used to evaluate the importance of each reference factor in the comprehensive evaluation. Therefore, the weighting coefficients of each factor can be determined by the ratio of the average of the correlation coefficients between a single factor and the other factors to the sum of the average of the correlation coefficients of all single factors. Firstly, the matrix of correlation coefficients between the four key factors of potato yield Y, starch content SC, water use efficiency WUE and systematic nitrogen use efficiency SNUE is required (**Table 4**); Secondly, by calculating the average value of correlation coefficients between each single participant factor and other single participant factors, r (**Table 5**), and the sum of the average values of correlation coefficients between all participant factors and other single factors, ∑r; and finally, by calculating the weight coefficients, r, to construct the weight coefficients matrix of each participant factor.

**Table 4:** Correlation coefficient matrix between the single evaluation factor of water and nitrogen regulation strategies for potatoes.

Participation indicators	Y (t ha^-1^)	SC (%)	WUE (kg m^-3^)	SNUE
Y	1			
SC	0.592	1		
WUE	0.982**	0.672*	1	
SNUE	0.409	0.486	0.377	1

Y indicates potato tuber yield, SC indicates the starch content of potato tubers, WUE indicates water use efficiency, and SNUE indicates system-based nitrogen use efficiency. * and ** indicate the effect significance at the 0.05 level, and the 0.01 level, respectively.

**Table 5:** Means of correlative coefficients among the factors in evaluation of the optimal water and nitrogen regulation strategies for potatoes and the significances of the factors.

	Y (t ha^-1^)	SC (%)	WUE (kg m^-3^)	SNUE
r	0.661	0.583	0.677	0.424
∑r	0.282	0.249	0.289	0.181

Y indicates potato tuber yield, SC indicates the starch content of potato tubers, WUE indicates water use efficiency, and SNUE indicates system-based nitrogen use efficiency. * and ** indicate the effect significance at the 0.05 level, and the 0.01 level, respectively.

According to **Table 5**, the weight coefficient matrix of each participant factor is constructed as follows:


$${\text{L}}_{1\times 4}=\left[\begin{array}{cccc} \text{Y} & \text{SC} & \text{WUE} & \text{SNUE}\\ 0.282 & 0.249 & 0.289 & 0.181 \end{array}\right]$$

#### 3.7.4 Calculation of integrated evaluation indicators

The product of the matrix of weight coefficients of the single participating factors and the single-factor judgment matrix is the integrated evaluation index for the optimal water and nitrogen regulation strategies of potato (IEI), and the matrix is constructed as follows:


$$\begin{array}{l} {\text{L}}_{1\times 4}\times {\text{M}}_{4\times 9}=(\begin{array}{cccc} 0.282 & 0.249 & 0.289 & 0.181 \end{array})\\ \times \left[\begin{array}{ccccccccc} \text{W}2\text{N}0 & \text{W}2\text{N}1 & \text{W}2\text{N}2 & \text{W}2\text{N}3 & \text{W}1\text{N}0 & \text{W}1\text{N}1 & \text{W}1\text{N}2 & \text{W}1\text{N}3 & \text{CK}\\ 0.523 & 0.671 & 0.847 & 0.926 & 0.571 & 0.698 & 0.951 & 1.000 & 0.579\\ 0.816 & 0.886 & 0.968 & 0.854 & 0.828 & 0.900 & 1.000 & 0.897 & 0.837\\ 0.604 & 0.761 & 0.911 & 0.966 & 0.655 & 0.754 & 1.000 & 0.961 & 0.612\\ 0.778 & 0.736 & 0.806 & 0.778 & 0.861 & 0.806 & 1.000 & 0.875 & 0.847 \end{array}\right]\\ =\left[\begin{array}{ccccccccc} \text{W}2\text{N}0 & \text{W}2\text{N}1 & \text{W}2\text{N}2 & \text{W}2\text{N}3 & \text{W}1\text{N}0 & \text{W}1\text{N}1 & \text{W}1\text{N}2 & \text{W}1\text{N}3 & \text{CK}\\ 0.666 & 0.736 & 0.889 & 0.894 & 0.712 & 0.785 & 0.987 & 0.934 & 0.702 \end{array}\right] \end{array}$$

#### 3.7.5 Optimal potato water and nitrogen control strategy selection

The results of the calculations indicate that the W1N2 treatment, characterized by a mild water deficit at the seedling stage and a nitrogen application of 185 kg ha⁻¹, achieved the highest IEI value of 0.987. This was followed by the W1N3 treatment, with an IEI value of 0.934, while the lowest IEI (0.666) was observed in the W2N0 treatment. Therefore, the W1N2 treatment was identified as the optimal water and nitrogen regulation strategy in this experiment. The W1N3 treatment was considered a viable alternative. The specific water deficit intervals, irrigation quotas at different growth stages, and nitrogen application rates and timing for these treatments are detailed in **Table 6**.

**Table 6:** Optimal water and nitrogen regulation strategies and their alternatives for potato.

Treatment	Indicator	Before sowing	Seeding period	Tuber formation period	Tuber expansion period	Starch accumulation period
W1N2	Soil moisture content (% of the field capacity)	–	55%-65%	65%-75%	65%-75%	65%-75%
	Irrigation quota (mm)	–	37.26	71.08	150.43	90.62
	Nitrogen application (kg ha^-1^)	111	–	74	–	–
W1N3	Soil moisture content (% of the field capacity)	–	55%-65%	65%-75%	65%-75%	65%-75%
	Irrigation quota (mm)	–	39.28	76.88	158.50	97.23
	Nitrogen application (kg ha^-1^)	144	–	96	–	–

## 4 Discussion

### 4.1 The impact of water-nitrogen regulation on potato growth

Plant height, stem thickness, and LAI are important indicators that reflect the adaptability of crops to environmental changes. Therefore, an accurate and efficient water-nitrogen management model is an effective way for potatoes to grow healthily and vigorously. However, potatoes are distinct from forage crops such as alfalfa. We pay more attention to their tuber yield. Therefore, water-nitrogen regulation measures should prevent excessive application of water-nitrogen fertilizer, which causes a large amount of redundant growth such as excessive plant height and leaf area, while also meeting the normal needs of potatoes. Thus, based on the theory of plant redundancy and excessive compensatory growth (Koch et al., 2020; Ierna and Mauromicale, 2023), this study found that W1N0 only had significant differences in plant height, stem thickness, and LAI with CK during the seedling (*p*<0.05), and both were significantly higher than CK at other growth periods (*p*<0.05), indicating that the W1 irrigation level did not significantly affect the growth and development of potatoes (Li et al., 2021a). In addition, at the W1 irrigation level, height, stem thickness, and LAI were significantly affected by nitrogen application rate, and growth indicators were highest at N3 level. However, the yield of W1N3 treatment was only 5.12 higher than W1N2 treatment (*p*>0.05). The aforementioned observation suggests that the aboveground part of potato plants at N3 application level may have indulged in excessive nitrogen absorption, leading to redundant growth of the aboveground part of the potato plant.

Combining dry matter accumulation (**Figure 4**), at the N0 application level, the potato had basically stopped growing at the end of the tuber swelling period, while N3 and N2 treatments applying nitrogen experienced rapid growth during the tuber expansion period. There was little difference in dry matter accumulation between W1N2 and W1N3 treatments, and both were not significantly different (*p*<0.05). However, the starch accumulation period further prolonged the growth process of potatoes under N3 treatment, resulting in dry matter accumulation of W1N3 treatment significantly higher than W1N2 treatment at the end of the growth period, followed by W2N3, but there was no significant difference between W1N2 and W2N3 treatment.

### 4.2 The impact of water-nitrogen regulation on potato yield and IWUE

Water and nitrogen are two important factors that affect crop yield and IWUE (Yang et al., 2023a; Yue et al., 2023). Previous studies have demonstrated that the utilization of potatoes can effectively conserve irrigation water and enhance IWUE when subjected to timely and moderate water stress, without causing a significant reduction in crop yield (Kifle and Gebretsadikan, 2016; Crosby and Wang, 2021; Ierna and Mauromicale, 2023). The findings of this current study align with these previous results, as it was observed that the W1 irrigation level led to an average increase in yield by 8.54% and average IWUE by 9.49% compared to the W2 irrigation level. Furthermore, the application of nitrogen fertilizer exhibited a positive correlation with both potato tuber yield and IWUE. The highest yield was 45.37 t ha^-1^ under W1N3 treatment, followed by W1N2 (43.16 t ha^-1^) (*p*<0.05). However, W1N2 had significantly higher IWUE (20.51 kg m^-3^) than W1N3 (19.26 kg m^-3^). The rationale behind this is that when subjected to N3 application level, potato growth exhibits the highest level of vigor, while its yield does not significantly differ from that of the N2 application level. The N3 application level is susceptible to excessive growth, resulting in a substantial consumption of water by potatoes, thereby rendering IWUE improvements insignificant.

### 4.3 The impact of water-nitrogen regulation on nitrogen balance

Nitrogen fertilizer input, as the main component of N_Input,_ affected soil inorganic N accumulation and supply. When comparing the inorganic N content in the soil at harvest to the content prior to sowing, all treatments demonstrated lower levels, with the exception for the N2 and N3 treatments, which exhibited a slight increase in the 0-20 cm soil layer (10.09%-16.74%). This suggests that nitrogen leaching loss did not occur in the present investigation. Notably, the absence of fertilizer led to a substantial decrease in soil residual N, which could potentially limit crop growth in the second season.

Plant N, as a major component of N_Output_, serves as a crucial metric for assessing the efficacy of nitrogen absorption and utilization. During the later stages of growth, potato tubers undergo expansion, resulting in an augmented proportion of tubers in dry matter accumulation. Consequently, tubers become the primary source of nitrogen uptake in potato plants. As a result, a maximum nitrogen uptake was recorded for the W1N3 treatment, followed by the W1N2 treatment, and it was not statistically significant that the two treatments differed (*p*<0.05). The average plant N under N0 conditions (INS = 103.96 kg N ha^-1^) closely approximated the average INS utilization in potato-producing regions in China (97.60 kg N ha^-1^) (Ning et al., 2023). However, owing to the constraints inherent in the employed methodology in this investigation, the absence of gaseous N monitoring, atmospheric deposition, biological N fixation, etc., the N_Output_ was significantly lower than the N_Input_, resulting in a N_unknow loss_ accounted for 17.00% to 28.65% of the N_Output_ in this study. Therefore, it is important to understand the mechanism and meaning of each possible source of N_unknow loss_ in the follow-up study to further explain the system-based SNUE.

### 4.4 The impact of water-nitrogen regulation on NUE indices

The NUE is influenced by different definitions and research perspectives (Congreves et al., 2021), leading to varying research results. Some studies have recorded significant changes in NUE indices in recent years (Conant et al., 2013; Lassaletta et al., 2014; Zhang et al., 2015). And research has found that the optimal water-nitrogen regulation mode for drip-irrigated potatoes in arid areas of Ningxia was an irrigation quota of 2100 m^3^ ha^-1^ and a nitrogen application rate of 110 kg ha^-1^ from the perspectives of yield, NPFP, and economic benefits. From the IWUE perspective, the optimal water-nitrogen regulation mode was an irrigation quota of 1200 m^3^ ha^-1^ and a nitrogen application rate of 270 kg ha^-1^ (Ning et al., 2023).

In this study, through the four perspectives of N fertilizer application, plant N uptake and utilization, soil inorganic N and N balance system, it was found that the W1N2 treatment had the highest NPFP, and NUE_crop_ (0.95), NUE_soil_ (0.94) and SNUE (0.74) were the closest to 1 in all the treatments, which indicated that the W1N2 treatment could increase crop productivity (NUE_crop_), maintain soil fertility (NUE_soil_) and improve nitrogen cycling system (SNUE). This discovery closely aligns with the previously determined optimal fertilization range established in the northwest region (Zhang et al., 2023), as well as the recommended value for the highest nitrogen application amount in Gansu Province based on the Nutrient Expert System (Jiang et al., 2023a).

### 4.5 The impact of water-nitrogen regulation on potato quality

Water and nitrogen are closely related to potato quality, in which the starch content and reducing sugar content of tubers determine its processing quality and technology, and vitamin C and protein content reflect the nutritional value of potato. Reasonable nitrogen application and irrigation are favorable to the increase of potato starch, vitamin C and protein content (Van Dingenen et al., 2019). The results of this study showed that potato starch, vitamin C and protein content with the increase of nitrogen application showed a trend of increasing and then decreasing, and reached the peak under N2 treatment (nitrogen application of 185 kg ha^-1^), which is consistent with the previous finding that the quality of potato tubers with the increase of fertilizer application showed the pattern of change of the single-peak curve (Wang et al., 2017). Reducing sugar content is an important index for high value-added processing of potato, and the lower the quality, the better. In this study, we found that the highest reducing sugar content was found in the treatment without nitrogen application, the reducing sugar content of potato tubers showed a decreasing trend with the increase of nitrogen application, and the lowest reducing sugar content was found in the N3 treatment (240 kg ha^-1^ of nitrogen application). The previous study also showed (Bi et al., 2020) that potato tuber reducing sugars under deficit irrigation showed a decreasing and then increasing trend with increasing fertilizer application, and the lowest reducing sugar content was found in the N application rate of 200 kg ha^-1^.

In addition, the previous study found that mild water deficit in the seedling stage had a small effect on potato quality (Li et al., 2021b), while water deficit in the tuber expansion stage and starch accumulation stage would reduce potato quality, and among them, potato starch, vitamin C and crude protein content increased with the increase of irrigation amount, and reducing sugar content decreased with the increase of irrigation amount (Zhang et al., 2023). This is consistent with the conclusion of this study that potato starch, vitamin C and protein contents were higher under mild water deficit at seedling stage than under moderate water deficit at seedling stage, while potato reducing sugar content was higher at W2 level than at W1 level.

### 4.6 Optimization of water and nitrogen regulation strategies for potatoes under drip irrigation under membrane

This study shows that mild water deficit in the seedling stage makes the post drought compensatory effect of potato significant, prompting the plant to transfer more photosynthetic products to the underground, resulting in a developed root system, which is more conducive to the later stage of the nutrients made in the above ground to the tubers, which is conducive to the increase in potato yield; and moderate water deficit in the seedling stage, due to the persistent soil moisture deficit in the seedling stage of the potato, resulting in the increase in the number of runners, which increases the number of potato The number of potatoes, but the later rewatering compensation effect can not make up for the long period of water deficit in the early stage of plant growth is affected, and thus the synthesis of photosynthesis products to reduce the biomass allocated to the underground tubers to reduce, so that the number of potatoes per plant increased, but the weight of potatoes per plant and the yield performance of the reduction.

In addition, no nitrogen plant growth dwarf, yellow leaves, photosynthetic product synthesis is limited, the nutrients allocated to the tubers to reduce the potatoes, so that serious yield reductions; while nitrogen application amount of nitrogen to the potato, the potato yield is reduced; and the potato yield is increased by the amount of nitrogen applied to the soil. Insufficient nitrogen application makes the aboveground part of the plant more weak stems, and the photosynthetic products are limited to the underground tuber transportation channel through the stalks, reducing the amount of nutrients to the underground tubers, which will also make the potato serious yield reduction; and excessive nitrogen application will result in the aboveground part of the plant growing too exuberantly and the underground tubers competing for nutrients, and the potato aboveground part of the greenery, and the underground part of the delayed potatoes and other undesirable phenomena, which will also cause the unknown nitrogen loss in the nitrogen system of the potato field to decrease, which will lead to a reduction in the yield of potato (Shrestha et al., 2023). It will also lead to the increase of unknown nitrogen loss (mainly gaseous nitrogen) in the nitrogen system of the potato field (Yang et al., 2024), which is not conducive to the realization of the goal of increasing potato yield and efficiency.

Therefore, in this study, the W1N2 treatment with seedling deficit regulation (mild water deficit at seedling stage and N application rate of 185 kg ha^-1^) was preferred as the optimal water and nitrogen regulation strategy for this experiment. This is basically consistent with the optimal water deficit regulation strategy of mild water deficit at seedling stage in the previous study (Li et al., 2021b), as well as the recommended N application threshold of 179-191 kg ha^-1^ for potato in northern China based on the nutrient expert system (Jiang et al., 2023b), which is closer to the optimal water and nitrogen regulation strategy of the previous study.

## 5 Conclusions

Water deficit and nitrogen application significantly impacted potato yield, quality, growth, development, water and nitrogen use efficiency, and soil nitrogen residue. To optimize the water and nitrogen management strategy, the integrated evaluation index for the optimal water and nitrogen regulation strategies of potato (IEI) was developed, incorporating key factors such as yield (Y), starch content (SC), water use efficiency (WUE), and systematic nitrogen use efficiency (SNUE). The results indicated that the W1N2 treatment, which involved a slight water deficit during the seedling stage and a nitrogen application rate of 185 kg ha^−1^, achieved the highest IEI score (0.987), making it the most effective water and nitrogen management strategy in this study. These findings offer a theoretical foundation for the rational management of water and nitrogen in potato cultivation within oasis irrigation regions, particularly for optimizing potato quality, yield, and efficiency.

## Data Availability

The original contributions presented in the study are included in the article/supplementary material. Further inquiries can be directed to the corresponding author.
